# Correlations between glycolysis with clinical traits and immune function in bladder urothelial carcinoma

**DOI:** 10.1042/BSR20203982

**Published:** 2021-02-19

**Authors:** Kai Che, Wenkai Han, Danxia Li, Shuxia Cui, Mingxin Zhang, Xiaokun Yang, Haitao Niu

**Affiliations:** 1 Department of Urology, The Affiliated Hospital of Qingdao University, Qingdao, China; 2 Department of Clinical Medicine, Qingdao University, Qingdao, China

**Keywords:** bladder urothelial carcinoma, clinical characteristics, glycolysis, immune activity

## Abstract

Background: Glycolysis was a representative hallmark in the tumor microenvironment (TME), and we aimed to explore the correlations between glycolysis with immune activity and clinical traits in bladder urothelial carcinoma (BLCA).

Methods: Our study obtained glycolysis scores for each BLCA samples from TCGA by a single-sample gene set enrichment analysis (ssGSEA) algorithm, based on a glycolytic gene set. The relationship between glycolysis with prognosis, clinical characteristics, and immune function were investigated subsequently.

Results: We found that enhanced glycolysis was associated with poor prognosis and metastasis in BLCA. Moreover, glycolysis had a close correlation with immune function, and enhanced glycolysis increased immune activities. In other words, glycolysis had a positive correlation with immune activities. Immune checkpoints such as IDO1, CD274, were up-regulated in high-glycolysis group as well.

Conclusion: We speculated that in BLCA, elevated glycolysis enhanced immune function, which caused tumor cells to overexpress immune checkpoints to evade immune surveillance. Inhibition of glycolysis might be a promising assistant for immunotherapy in bladder cancer.

## Introduction

Bladder urothelial carcinoma (BLCA) is one of the most common urinary malignancies, ranking as the eleventh most commonly diagnosed cancer worldwide. At the same time, it rises to seventh when only males are considered [1]. Based on data from World Health Organization (WHO), approximately 549400 people were diagnosed with BLCA, and 199900 died of this cancer in 2018. Thus, it is a great threat to human health [2]. Up to 25% of patients were identified as muscle-invasive bladder cancer (MIBC) for the first diagnosis, which usually leads to distant metastasis and dismal prognosis [3]. Although radical cystectomy is the standard treatment for MIBC, the U.S. Food and Drug Administration (FDA) granted approvals to two immune checkpoint inhibitors (ICIs), pembrolizumab and atezolizumab, for first-line treatment of cisplatin-ineligible patients with PD-L1-positive bladder cancer in 2018 [4]. Due to its limited application scope, new systemic therapeutic approaches are urgently needed for BLCA patients. Exploring the interaction between tumor cells and tumor microenvironment (TME) could lead to a deeper understanding of BLCA initiation, progression, and metastasis, possibly providing prospective cancer prevention and treatment strategies.

One hallmark of cancer is reprogramming energy metabolism [5], of which aerobic glycolysis is the most representative in TME. Aerobic glycolysis, also known as the ‘Warburg effect’, is a general way of glucose metabolism in cancer cells. In this way, cancer cells tend to produce energy via aerobic glycolysis rather than tricarboxylic acid cycle, even in the presence of oxygen [6]. Glycolysis not only supplies energy for cancer cells, but also provides intermediate products for various biosynthetic pathways. Studies have shown that glycolysis is a promising target for cancer therapy [7]. It is associated with immune escape from cancer [8], and inhibition of glycolysis can fight against cancer. In BLCA, PDK1, a glycolytic enzyme, was correlated with poor prognosis [9]. Moreover, another enzyme, PKM2, plays an essential role in cell proliferation and cell cycle. The down-regulation of PKM2 suppressed proliferation of bladder cancer cells, with a decreased proportion of cells in the DNA synthesis phase [10]. Glycolysis plays a critical role in bladder cancer.

Except for tumor cells, there are various immune cells in TME, including macrophage subtypes, mast cells, neutrophils, and myeloid-derived suppressor cells, as well as T and B lymphocytes [11]. It is generally held that immune cells infiltration in TME could interplay with tumor cells, which may contribute to tumor progression or inhibit the efficacy of existing anti-cancer therapies [12]. Immune cells in TME have dual characters, some might be pro-tumor, while others are anti-tumor [13]. Prior studies found that enhanced glycolysis impairs T cell killing of tumor cells and induces pro-tumor immunity. Glycolytic activity was a prospective predictor of immune signatures in diverse cancers [14]. Correlations between glycolysis and immune activity have not been explained in BLCA, and exploration of these would provide a broader view of biological processes, contributing to finding more effective therapeutic strategies.

In our study, we used bioinformatics tools to explore the associations between glycolysis and immune activity by analyzing the TCGA database. We used a single-sample gene set enrichment analysis (ssGSEA) algorithm to grant a glycolytic score for each sample based on a glycolytic gene set. So, BLCA patients can be divided into a high-glycolysis group (glycolysis-H) and a low-glycolysis group (glycolysis-L). We analyzed the differentially expressed genes (DEGs) between two groups, and enrichment analyses were conducted. We found that the level of glycolysis was positively related to immune activity. Enhanced glycolysis might contribute to distant metastatic and immune escape as well.

## Materials and methods

### Materials

We obtained mRNA transcriptome data of BLCA from TCGA, which was downloaded directly from TCGA data portal (https://portal.gdc.cancer.gov/). The downloaded data consisted of 409 tumor samples, which were used for further research. The corresponding clinicopathological features including age, gender, primary tumor, regional lymph node, distant metastasis, tumor stage, grade, survival time, and survival status, were also obtained from TCGA data portal.

### Evaluation of tumor glycolysis score

Based on HALLMARK-GLYCOLYSIS, KEGG-GLYCOLYSIS-GLUCONEOGENESIS, and REACTOME-GLYCOLYSIS from the Molecular Signature Database (https://www.gsea-msigdb.org), we extracted a gene set of glycolysis (Supplementary Table S1). Gluconeogenesis-related genes were excluded and some genes enhancing glycolysis activity such as glucose transport (encoded by SLC2A), monocarboxylic acid transporter (encoded by SLC16A) were added to the set. Based on the glycolysis gene set, an ssGSEA algorithm was applied to quantify each sample’s glycolytic score. We defined a group as highly glycolytic (glycolysis-H) if its glycolysis score was in the upper half of all glycolysis scores. Another group was lowly glycolytic (glycolysis-L) if its glycolysis score was in the bottom half.

### Identification of DEGs and functional enrichment analysis

The tumor samples were divided into glycolysis-H group and glycolysis-L group based on the median of the glycolysis score. Gene expression profiles were derived based on differential genes expression analysis using the limma R package. The thresholds for DEGs were |log2FC| ≥ 1 and adjusted *P*-value <0.05. Gene ontology (GO) analysis and Kyoto Encyclopedia of Genes and Genomes (KEGG) analysis were performed using *P*<0.05 as the cut-off criterion. Gene set enrichment analysis (GSEA) was conducted to find significantly up-regulated and down-regulated pathways between the two groups using FDR < 0.05 (http://www.webgestalt.org/). Besides, differences in survival and clinicopathological features between glycolysis-H group and glycolysis-L group were explored.

### Evaluation of immune signature enrichment

Based on 29 immune gene sets (obtained from an excellent study [15], Supplementary Table S2), including genes related to different immune cell types, functions, pathways, and checkpoints, ssGSEA algorithm was employed to comprehensively evaluate the immunological characteristics of each sample included in the study. Twenty-nine immune scores were acquired, and then they were applied to identify correlations with glycolysis score via Spearman correlation.

### Statistical analysis

R 3.6.2 and SPSS 23.0 were utilized for statistical analysis. The overall survival (OS) rate of each group was estimated using the Kaplan–Meier method, and differences between groups were assessed by the log-rank test. Categorical data were compared using the chi-square test or Fisher’s exact test, while enumeration data were conducted via *t* test. For statistical purposes, some groups are merged. Correlations between immune scores and glycolysis scores were calculated using Spearman correlation. *P*-value <0.05 was considered statistically significant.

## Results

### Glycolysis score can effectively reflect glycolysis activity

A glycolysis gene set was extracted from three glycolysis gene sets, including HALLMARK-GLYCOLYSIS, KEGG-GLYCOLYSIS-GLUCONEOGENESIS, and REACTOME-GLYCOLYSIS from Molecular Signature Database [16]. A total of 58 genes were included, which can encode enzymes and metabolite transporters involved in glycolysis. Based on the set, glycolysis scores for each sample (Supplementary Table S3) were quantified using the GSVA R package [17].

**Figure 1:**
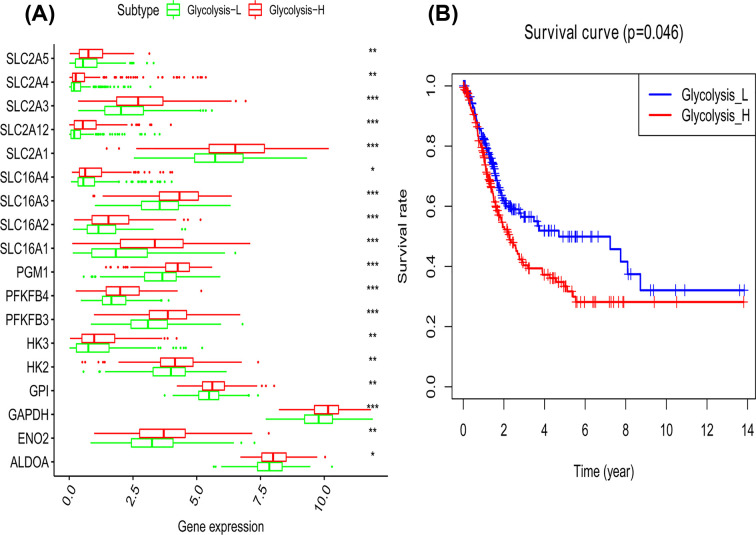
Glycolysis scores effectively reflected glycolysis activity and was related to poor prognosis (**A**) Up-regulated glycolytic genes in glycolysis-H group; (**B**) enhanced glycolysis was associated with poor prognosis in bladder cancer.

For further analysis, we explored whether glycolysis score could represent glycolytic activity. We divided BLCA patients into glycolysis-H group and glycolysis-L group by median score, then DEGs were screened out. We found that in glycolysis-H group, numerous glycolysis-related genes such as SLC2A5, SLC2A4, SLC2A3, SLC2A12, SLC2A1, SLC16A4, SLC16A3, SLC16A2, SLC16A1, PGM1, PFKFB3, PFKFB4, HK2, HK3, GPI, GAPDH, ENO2, ALDOA, had varying elevation (Figure 1A). These up-regulated genes mainly encode glycolytic enzymes, glucose transporters (GLUTs), and monocarboxylic acid transporters (MCTs), which were beneficial to tumor cells’ glycolysis. In addition to enhanced glycolytic enzymes, GLUTs were up-regulated to meet their increased glucose demand [18], and MCTs were elevated to facilitate tumor cells to expel excessive lactic acid within cells [19]. In these ways, glycolytic activity was remarkably enhanced. All these indicated that glycolysis-H group had higher glycolytic activity than glycolysis-L group, and our classification method was reliable. The score can reflect glycolytic activity to some extent.

### Glycolytic activity is associated with clinical characteristics in BLCA

We firstly explored the association between glycolytic score and prognosis in BLCA. Survival analysis showed that glycolysis-H group had shorter OS (Figure 1B), which revealed the prognostic significance of glycolytic activity. It is also confirmed by our analysis for survival status between the two groups (Table 1). Previous studies have come to consistent conclusions. Using a glycolysis-based 4-mRNA signature, they found that high-risk group had higher mortality and poorer OS than low-risk group in bladder cancer [10]. It was generally held that glycolysis was related to poor prognosis in cancers, and the reliability of glycolysis score was demonstrated in another way.

**Table 1:** Correlations between clinical characteristics and glycolytic activity in bladder cancer

Characteristics	Glycolysis-H	Glycolysis-L	*P*-value
Age	68.69 ± 10.197	67.49 ± 10.996	0.256
Gender (F/M)	49/155	56/145	0.428
T stage			0.724
T1	2	1	
T2	55	64	
T3	102	90	
T4	30	28	
TX	15	18	
N stage			0.651
N0	121	114	
N1	23	23	
N2	36	39	
N3	2	6	
Nx	22	19	
M stage			0.003
M0	81	113	
M1	6	5	
Mx	117	83	
AJCC stage			0.243
I+II	60	71	
III+IV	144	130	
Grade			0.121
High	197	187	
Low	7	14	
Survival status			0.012
Live	102	126	
Dead	102	75	

Correlations between other clinical characteristics and glycolysis score were also disclosed. Although we found no differences in age, gender, T stage, N stage, AJCC stage, grade between glycolysis-H group and glycolysis-L group, the difference in M stage was obvious (Table 1), which revealed that enhanced glycolytic activity facilitated invasion and metastasis in BLCA. This phenomenon was also revealed in pancreatic cancer [20], breast cancer [21], hepatocellular carcinoma [22], and gastric cancer [23].

### Glycolytic activity was closely related to immune function

**Figure 2:**
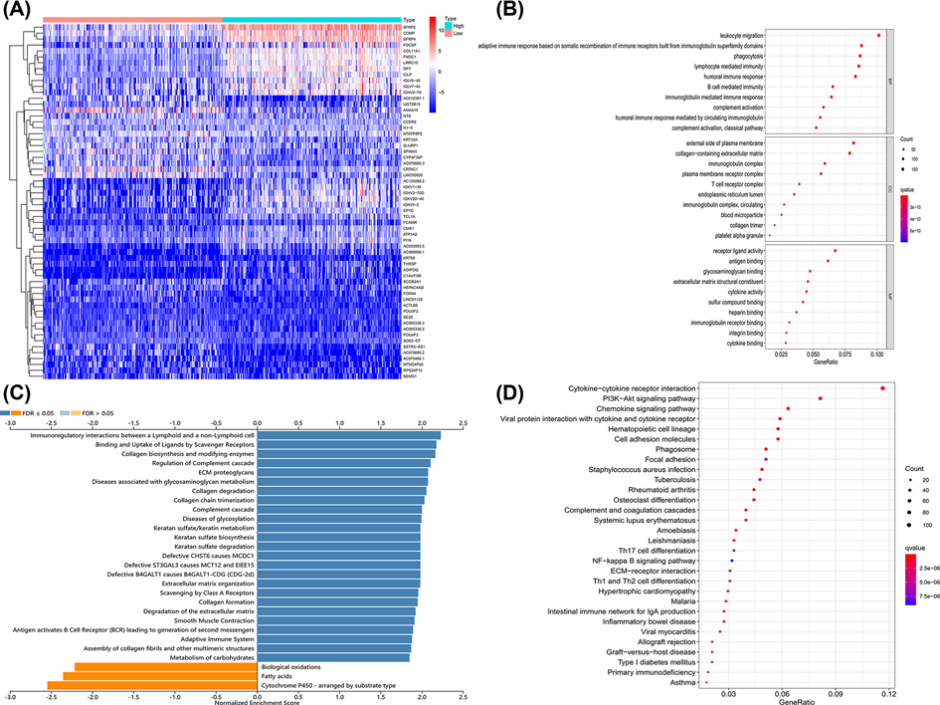
Glycolysis was closely related to immune function (**A**) Top-30 up-regulated and down-regulated genes between glycolysis-H and glycolysis-L; (**B**) GO annotion based on DEGs; (**C**) enrichment analysis of REACTOME pathways by GSEA; (**D**) KEGG enrichment analysis according to DEGs.

To further explore the biological difference between glycolysis-H group and glycolysis-L group, we analyzed DEGs (Supplementary Table S4). A total of 2856 genes were identified, including 2234 up-regulated and 622 down-regulated genes, to be significantly related to glycolysis. In the top 30 up-regulated genes, we found that several genes encoding immunoglobulin were up-regulated in glycolysis-H group and involved in the recognition phase or effector phase of humoral immunity (Figure 2A), which indicated that glycolysis and immune function might be closely related.

GO (Figure 2B) and KEGG (Figure 2D) enrichment analysis were conducted to reveal the gene function and biological pathways of DEGs. Our results indicated that glycolytic activity was closely related to immune function. To be more specific, the immune-related activities included leukocyte migration, adaptive immune response based on somatic recombination of immune receptors built from immunoglobulin superfamily domains, phagocytosis, lymphocyte-mediated immunity, humoral immune response, B cell-mediated immunity, immunoglobulin-mediated immune response, complement activation, humoral immune response mediated by circulating immunoglobulin, classical pathway of complement activation, immunoglobulin complex, T cell receptor complex, circulating immunoglobulin complex, receptor ligand activity, etc. The enriched pathways were cytokine−cytokine receptor interaction, chemokine signaling pathway, viral protein interaction with cytokine and cytokine receptor, *Staphylococcus aureus* infection, tuberculosis, rheumatoid arthritis, complement and coagulation cascades, Th17 cell differentiation, Th1 and Th2 cell differentiation, primary immunodeficiency, etc. Similar enrichment results of REACTOME pathways analysis were obtained by GSEA analysis, with the most relevant positive pathway being immunoregulatory interactions between a lymphoid and a non-lymphoid cell (Figure 2C).

### Glycolysis had positive correlations with immune activity

**Figure 3:**
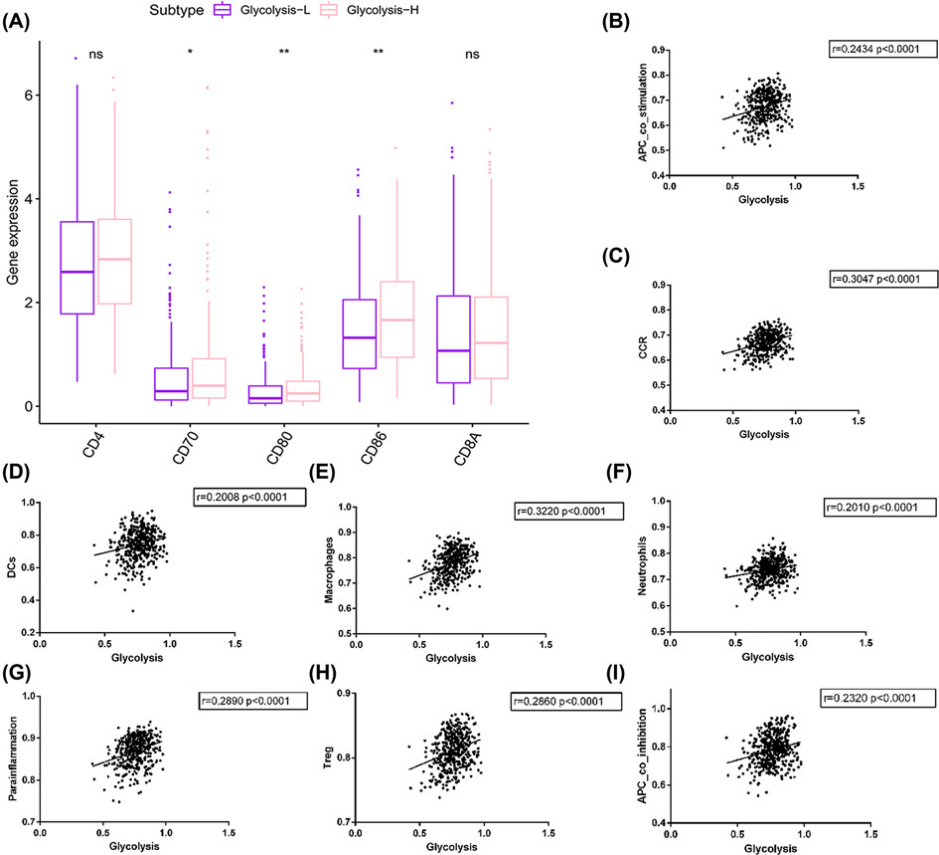
Glycolysis was positively associated with immune activities (**A**) Several immune-related genes were up-regulated in glycolysis-H group. (**B–I**) Glycolytic score had positive correlation with APC_co_stimulation, CCR, DCs, Mcrophages, Neutrophils, parainflammation, Treg and APC_co_inhibition, respectively. Abbreviations: APC, antigen presenting cell; CCR, chemokine receptor; DC, dendritic cell. *, *P*<0.05. **, *P*<0.01. ***, *P*<0.001.

As we found in the biological process, leukocyte migration was the most enriched biological process. We speculated that enhanced glycolysis would attract leukocytes to TME. In the DEGs profile, we found that numerous CC motif chemokine ligand (CCL) genes were significantly up-regulated (Supplementary Table S5). These genes encode chemotactic factor that attracts naive T cells, CD4^+^ and CD8^+^ T cells [24], monocytes, resting T lymphocytes and neutrophils [25]. Moreover, genes involved in T-cell activation, the induction of cell proliferation and cytokine production, and promotion of T-cell survival were elevated in glycolysis-H group, such as CD28, CD80, CD70 (Figure 3). The surface marker proteins of CD4^+^ and CD8^+^ T cells, CD4, and CD8A were up-regulated as well, though no statistical significance was found. All these indicate that glycolysis could lead to aggregation of immune cells and activate them to function.

Subsequently, correlations between glycolytic score with 29 immune signature scores (Supplementary Table S6) were explored as well. Our analysis showed that, to some extent, glycolytic score was positively related with antigen presenting cell (APC) co-stimulation, chemokine receptor (CCR), dendritic cells (DCs), macrophages, neutrophils, parainflammation, Treg and APC co-inhibition signatures (Figure 3). In a word, glycolytic score had positive correlations with diverse immune activities.

### Tumor immune evasion was activated in glycolysis-H group

We found a paradox that high glycolysis score was positively related to immune activity [14], meanwhile, it was also associated closely with distant metastasis and poor OS. Therefore, we hypothesized that high glycolysis might lead to tumor cells’ resistance to immune function, resulting in immune evasion. To verify our guess, we investigated tumor-related immune checkpoints, including CD273, CD274, CTLA-4, and the ones that were newly emerging, LAG-3, TIM-3, TIGIT, CD276, BTLA, and IDO1 [26], between the two subgroups.

**Figure 4:**
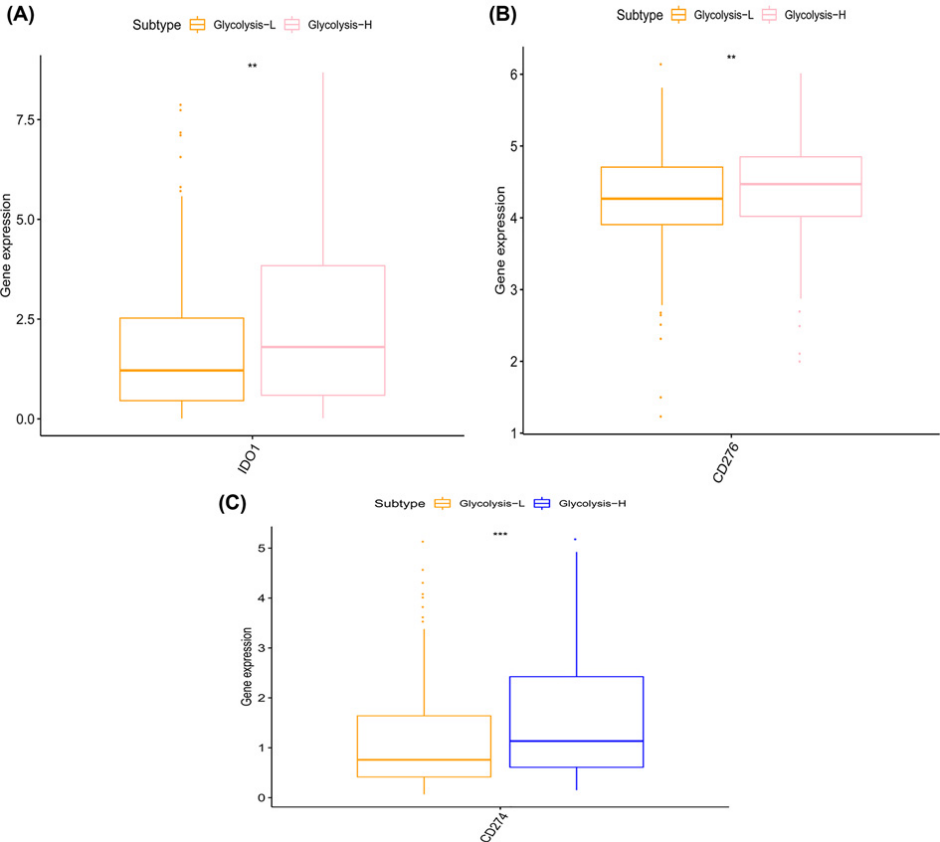
Immune checkpoints were elevated in glycolysis-H group IDO1 (**A**), CD276 (**B**), and CD274 (**C**) were up-regulated in glycolysis-H group, respectively. *, *P*<0.05. **, *P*<0.01. ***, *P*<0.001.

We found that CD274, CD276, and IDO1 have varying elevations in glycolysis-H group (Figure 4). It was reasonable to speculate that increased glycolysis could enhance immune function, thus activating immune escape of tumor cells. Similar conclusions were obtained in pan-cancers research that tumor glycolysis increases tumor immunity and enhances PD-L1 expression [14].

## Discussion

In recent years, as a hallmark of cancer, glycolysis has attracted much attention. Tumor cells mainly rely on aerobic glycolysis as a major source of energy and intermediate products for their uncontrolled growth and proliferation, so do BLCA cells [27]. It had been proved that increased aerobic glycolysis contributed to tumor aggressiveness and was associated with poor prognosis in BLCA [28]. However, most of the previous studies on glycolysis merely focused on glycolytic enzymes. In our study, 58 glycolysis-related genes, modified by manual based on three known glycolysis gene sets, were used to acquire glycolysis score by ssGSEA for each sample. According to the glycolysis score, we divided BLCA samples into glycolysis-H group and glycolysis-L group. In glycolysis-H group, we observed that in addition to some glycolytic enzymes, GLUTs and MCTs were elevated to varying degrees.

GLUTs, encoding by the solute carrier 2 family (SLC2) genes, were regarded as one of the most critical proteins controlling glycolytic flux [29]. Tumor cells consume much more glucose than normal cells, then to meet the demand, GLUTs were up-regulated to increase the glucose uptake ability. The up-regulation of various glucose transporters, primarily GLUT1, 3, and 4, was observed in endometrial cancer [30], gastric cancer [31], squamous cell carcinomas [32], meningiomas [33], and glioblastomas [34], which was found in glycolysis-H group in our study as well. Previous studies have confirmed that the expression of GLUT1 in human bladder cancer is associated with poor prognosis and a low survival rate [35], which was considered a marker of aggressive biologic potential for BLCA meanwhile [36]. Besides, suppressing GLUT3 in BLCA could inhibit cell growth and promote apoptosis [37]. We concluded that increased GLUTs could reflect elevated glycolytic activity to some extent, and it might be closely associated with poor prognosis in BLCA.

As most tumors depended on glycolysis for energy production, numerous lactates were produced. MCTs subsequently were up-regulated to mediating lactate efflux and pH. MCTs, especially MCT1 and MCT4, were important contributors to tumor aggressiveness and poor prognosis [38], which had been verified in colorectal carcinoma [39], gastric carcinoma [40], and esophageal adenocarcinomas [41]. In BLCA, once MCT1 was knocked out, the proliferation, migration, and invasion of bladder cancer cells were inhibited [42]. We concluded that increased MCTs would promote tumor invasiveness to facilitate distant metastasis and were related to poor prognosis. These indicated that both GLUTs and MCTs facilitated glycolysis, which had correlations with poor prognosis and metastasis.

So far, much work has been focused on tumor glycolysis. However, the biological events affected by tumor glycolysis, especially immune activity, have not been well disclosed in BLCA. We divided the samples into glycolysis-H group and glycolysis-L group according to glycolysis score, subsequently screened DEGs profile, and conducted functional enrichment analysis. The results suggested that large amounts of immune activities were involved, indicating that glycolytic activity is closely related to immune function in bladder cancer. Furthermore, numerous cytokines, chemokines, related receptors, and immune activation-related genes were up-regulated in the high glycolysis group. Significant positive correlations between glycolytic activity and various immune activities in bladder cancer were found. Previous research on pan-cancer reached an agreement with us [14]. Li et al. explored the association between glycolysis and the immune cells. They concluded that most of the immune cell type enrichment scores were remarkably higher in the high glycolysis group, including CD8^+^ naive T cells, Th1 cells, Th2 cells, NK cells, Monocytes, Macrophages M1, DC, and so on [43]. Taken together, glycolysis activity was closely related to immune activity, and immune function was activated as glycolysis was elevated in bladder cancer.

What confused us was that the up-regulated immune activity did not bring a good outcome to the patients in glycolysis-H group. Several studies have revealed that tumor cells’ metabolic microenvironment, including lactic acid accumulation and acidification of TME, caused by glycolysis, may selectively disable tumor-killing T and NK cell activation and promote immune evasion [44,45]. Similar results were found in our study that IDO1, CD274, CD276 were up-regulated in glycolysis-H group. One explanation would be that increased glycolysis enhances the immune system’s ability to kill tumor cells so that they up-regulated immune checkpoint to survive in such a tough environment.

In recent years, immunotherapy, especially anti-PD-1/PD-L1 therapy, has become a research hotspot in cancer therapy. Due to limited effectiveness, the application of immunotherapy was restricted. Tumors with high glycolysis are more likely to respond to anti-PD-1/PD-L1 therapy than tumors with low glycolysis. The more active response in highly glycolytic tumors may be owing to the elevated PD-L1 expression. We speculated that, combined with immunotherapy, inhibition of glycolysis might be a promising therapeutic target for bladder cancer. Certainly, further studies were necessary to confirm this viewpoint.

## Conclusion

In conclusion, increased glycolysis was associated with poor prognosis and might facilitate distant metastasis in bladder cancer. Studies on GLUTs and MCTs also confirm this result. Glycolysis was closely related to immune activities, and increased glycolysis enhance immune function to kill tumor cells. As a result, tumor cells might up-regulate immune checkpoints to survive from immunological surveillance. Inhibition of glycolysis might be a promising combination therapy for immunotherapy. Further studies were needed to confirm our hypothesis.

## Supplementary Material

Supplementary Tables S1-S6

## Data Availability

We obtained mRNA transcriptome data of BLCA from TCGA, which was downloaded directly from TCGA data portal (https://portal.gdc.cancer.gov/).

## Abbreviations

AJCC: American Joint Committee on Cancer
APC: antigen presenting cell
BLCA: bladder urothelial carcinoma
DC: dendritic cell
DEGs: differentially expressed genes
FDR: false discovery rate
GLUT: glucose transporter
GO: Gene Ontology
GSEA: gene set enrichment analysis
KEGG: Kyoto Encyclopedia of Genes and Genomes
MCT: monocarboxylic acid transporter
MIBC: muscle-invasive bladder cancer
OS: overall survival
PD-L1: Programmed Cell Death Ligand 1
PD-1: programmed cell death protein 1
SLC2: solute carrier 2 family
ssGSEA: single-sample gene set enrichment analysis
TCGA: The Cancer Genome Atlas Program
TME: tumor microenvironment
Treg: Regulatory cells
